# Optimisation of the sampling method for skin microbiome studies in healthy children: a pilot cohort study

**DOI:** 10.3389/frmbi.2024.1446394

**Published:** 2024-11-12

**Authors:** Anita Smith, Noor-Ul-Huda Ghori, Rachael Foster, Mark P. Nicol, Timothy Barnett, Janessa Pickering, Alexandra Whelan, Tobias Strunk, Fiona Wood, Edward Raby, Mark Fear, Stephanie Weston, Anita J. Campbell, Gerard F. Hoyne, Asha C. Bowen

**Affiliations:** ^1^ Wesfarmers Centre of Vaccines and Infectious Diseases, The Kids Research Institute Australia, Perth, WA, Australia; ^2^ Department of Dermatology, Perth Children’s Hospital, Perth, WA, Australia; ^3^ School of Health Sciences, University of Notre Dame, Fremantle, WA, Australia; ^4^ Department of Dermatology, Fiona Stanley Hospital, Perth, WA, Australia; ^5^ Division of Infection and Immunology, School of Biomedical Sciences, The Marshall Centre, The University of Western Australia, Perth, WA, Australia; ^6^ Neonatal Directorate Child and Adolescent Health Service, King Edward Memorial Hospital for Women, Subiaco, WA, Australia; ^7^ Department of Plastic Surgery, Perth Children’s Hospital, Perth, WA, Australia; ^8^ Department of Plastic Surgery, Fiona Stanley Hospital, Perth, WA, Australia; ^9^ Department of Infectious Diseases, Fiona Stanley Hospital, Perth, WA, Australia; ^10^ Department of Infectious Diseases, Perth Children’s Hospital, Perth, WA, Australia; ^11^ Institute for Respiratory Health, The University of Western Australia, Nedlands, WA, Australia; ^12^ Menzies School of Health Research, Charles Darwin University, Darwin, NT, Australia

**Keywords:** skin, microbiome, 16S rRNA sequencing, FLOQ swab, skin scraping, paediatrics, dermatology

## Abstract

**Introduction:**

Recent interest in the diverse ecosystem of bacteria, fungi and viruses that make up the skin microbiome has led to numerous studies investigating the skin microbiome in healthy skin and in dermatological conditions. However, skin microbiome analysis is challenging due to relatively low numbers of skin microorganisms compared to mucosal sites, such as the respiratory or gastrointestinal tracts. Microbiome results are heavily influenced by sampling methods. Previous sampling methods include that of cotton swabs, tape stripping, patch sampling and punch biopsies. It is essential to have a standardised sampling method for microbiome studies to have comparable results between studies. Two non-invasive methods of sampling the skin microbiome; a skin scraping versus a flocked swab were chosen as methodologies likely to be efficient, effective, and easy to access for future skin microbiome studies in children. Here we compare the two sampling methods to describe the composition of the skin microbiome in healthy children.

**Method:**

Samples were collected from six healthy children aged three to nine years from the skin overlying the cubital fossa, cheek and axilla using (i) flocked swabs and (ii) skin scrapings with a glass slide. Samples were collected from the left and right sides of the body at two separate time points, one week apart. Quantitative PCR of the gene encoding 16S ribosomal ribonucleic acid (rRNA) was performed to compare the bacterial load collected by each sampling method. Full-length 16S rRNA gene amplicon sequencing was performed to compare the relationship of sampling method and time with the diversity and ecology of bacteria between different body sites.

**Results:**

From six children, 78 flocked swabs and 78 skin scraping samples were collected, along with details of their overall health and skin care practices. qPCR results indicated higher total bacterial load from flocked swabs compared with skin scrapings. Flocked swabs and skin scraping methods had very similar bacterial compositional profiles. The skin microbiome was diverse between individuals and remained relatively stable within individuals over time.

**Discussion:**

Overall, results were similar between sample types, however bacterial DNA yield was higher for flocked swab samples (compared to skin scraping methods) and with a simpler protocol is the preferred sampling method for future studies.

## 1 Introduction

The skin microbiome is a diverse ecosystem of bacteria, fungi, and viruses, however, compared to other body sites, it has a very low biomass due to its nutrient poor, exposed and dry environment (Chen et al., 2018; Chen et al., 2023). Whilst microbiome research is advancing rapidly, the skin microbiome represents unique challenges due to the low biomass environment. Most available protocols for microbial characterisation are based on high biomass, high diversity gut microbiome (Bjerre et al., 2019). Low biomass samples are susceptible to contamination from environmental sources during processing leading to false positive findings, and have an increased likelihood of other biases (Greathouse et al., 2019; Eisenhofer et al., 2019; Salter et al., 2014).

Until as recently as twenty years ago, methods of investigating human skin microbes used culture based techniques (Chen et al., 2023). These initial studies identified primarily *Staphylococcus*, *Cutibacterium* (formerly *Propionibacterium*), *Corynebacterium*, and fungi such as *Malassezia* (Chen et al., 2023; De Pessemier et al., 2021). However, not all microbes on the skin can be cultured *in vitro* and some do not survive removal from the skin, creating bias, with the microbial richness of the skin being underestimated (Santiago-Rodriguez et al., 2023; Chen et al., 2023). The advantage of 16S ribosomal ribonucleic acid (rRNA) gene sequencing includes the ability to reveal the presence of diverse bacterial phyla, the ability to study the microbiome of particular skin diseases, the low cost compared to other sequencing methods and the ability to avoid sequencing host DNA (Santiago-Rodriguez et al., 2023). However, short-read amplicon based sequencing is unable to provide accurate information about species or strains of microorganisms on the skin (Chen et al., 2023) and is highly dependent on sampling methods. These, in turn, are influenced by multiple factors such as the multiple layers of the skin and uneven species distribution (Bjerre et al., 2019).

Several sampling methods for investigating the skin microbiome have been described (Grice et al., 2008; Manus et al., 2020; Alyami et al., 2023). Cotton swabs and skin scrapings have been used to obtain comparative skin microbiome profiles, representative of those obtained with skin biopsies, a technique often used in dermatology clinical practice to further evaluate for deeper skin infection and disease (Grice et al., 2008). Tape stripping and scraping have also been reported in the literature but with suboptimal microbiome results (Alyami et al., 2023). Another method considered is adhesive patch sampling, which has been reported to be effective, well tolerated and non-invasive. However, adhesive patch-based skin biopsy devices are difficult to procure. More invasive approaches include skin punch biopsies which have been used to analyse the follicular skin microbiome using 16S rRNA and 18S rRNA sequencing (Ring et al., 2017). Disadvantages with punch biopsies include that they are an invasive procedure that usually require suturing, can leave a scar, which may be problematic if wanting to sample sites on the face (forehead, nose) and may not be appropriate for sensitive sites (such as axillae or groin) (Pistone et al., 2021) or for repeated longitudinal sampling.

Compared to cotton swabs, flocked swabs have been shown to generate superior DNA extraction yields, and are more suitable for direct PCR (Toohey-Kurth et al., 2020; Van Horn et al., 2008; Wise et al., 2021). The tip of a flocked swab is like a brush, creating a greater surface area compared to cotton swabs and an ability to collect more material. The brush like tip also enables superior specimen collection and release during testing. However, differences in frequency of swabbing, direction of swabbing, and pressure when swabbing are difficult to control and can alter the output in terms of viable bacteria and DNA concentration upon extraction (Van Horn et al., 2008). Manus et al. (2020), analysed 16S rRNA bacterial gene sequences from swab (dry sterile media-free cloth swab) samples taken from the axilla, hand, and forehead of 47 infants and found the bacterial diversity and composition are shaped by skin site, age, socioeconomic factors, and household composition. This study showed that predominantly *Proteobacteria*, *Firmicutes*, *Bacteroidetes* and *Actinobacteria* were reported in skin samples from two age groups (0 to 6 months and 7 to 33 months). However, overall there is no established standard sampling method that produces unbiased results for skin microbiome studies.

Hence, further studies to optimise the molecular detection of bacteria from skin with standardised methods for sampling (Chen et al., 2023) are required to inform a broader understanding of the skin microbiome in health and disease. In this study, we aimed to optimise sampling of the skin microbiome by comparing two frequently used strategies, namely flocked swabs, and skin scrapings. To establish our findings across different body sites, we sampled the cubital fossa, cheek and axilla, representing different skin types, and re-sampled the same participants one week after the first sampling. We used full length 16S rRNA sequencing to achieve species-level discrimination of the bacterial microbiome.

## 2 Materials and methods

### 2.1 Study design

This was an observational cohort study following methodology outlined by the STROBE statement (von Elm et al., 2007). We aimed to develop a sampling protocol for skin microbiome analysis.

### 2.2 Study participants

Healthy child volunteers were recruited via the immunisation clinic at the Perth Children’s Hospital (PCH) in Perth, Western Australia in January 2021. This study was approved by the ethics committee of the Child and Adolescent Health Service (RGS – 3841) and University of Notre Dame Cross Institutional Approval (2021 – 127F). Prior to sampling, parents completed a short online questionnaire. Participants were recruited over a two-week period and sampled in the dermatology outpatient clinics, with sampling and questionnaire at the time of sampling and one week later. Inclusion criteria included healthy children aged 1–10 years, English speaking, and whose parent/guardians were able to provide written informed consent for participation. Participants who reported to have current open skin/wounds/current skin infection in sites requiring sampling (cubital fossa, face and axilla), current health issues (i.e. current inpatient, current exacerbation of chronic condition or acute health issues), use of any creams/ointments/preparations regularly on their skin (including topical steroids), currently receiving or having received antibiotic/antifungal/antiviral treatment of any form (IV, oral, topical) in the past two weeks and children who had used antiseptic/antimicrobial topical preparations in the past 48 hours on skin sites for sampling were excluded. Families were instructed to avoid moisturisers, sunscreens, or creams on the child’s skin in the morning of areas being sampled, avoid swimming in a chlorinated pool or ocean in the 24 hours prior to sampling and were advised not to apply any topical antiseptics on the skin in the 48 hours prior to sampling. Samples were taken from the skin overlying the axillae, cheek, and cubital fossa body sites. As this was an early phase study designed to collect information on skin microbiome complexity and variability, and to determine a method, no power calculation was performed to determine study size. Therefore, this study was not statistically powered to detect changes in minor bacterial populations.

### 2.3 Sample collection and storage

Skin samples were collected from non-overlapping areas on the cubital fossae, cheek, and axillae from the left and right sides of the body. The composition of the skin microbiome varies depending on the moisture content, temperature and sebaceous gland concentration in addition to other factors such as the exogenous environment and host genetics (Griffiths et al., 2016). These can include the sebaceous (oily) zone (e.g., forehead), the dry zone (e.g., volar forearm), and the moist zone (e.g., antecubital fossa, axilla). Specifically, the sites for this study were chosen to represent zones in the skin microbiome and to represent various diseases that are influenced by the skin microbiome e.g. atopic dermatitis (cubital fossa) and hidradenitis suppurativa (axilla). Areas for sampling were marked using a 5 cm^2^ square stencil template. Two doctors completed the sampling, with one starting with the flocked swab (FLOQÒ, COPAN, Murrieta, CA, USA) first, then the scraping, and the second starting with the scraping first, followed by flocked swab. Two doctors performed the sampling per child simultaneously with one operator sampling the left side and another operator sampling the right side simultaneously. Samples were collected in order: initially from the left cubital fossa, right cubital fossa, left cheek, right cheek, left axilla then right axilla, giving a total of 6 swab samples and 6 scraping samples per visit for each child. Two control samples were also collected at the start and end of the sampling sessions (details below). For sample collection, no prior cleaning or preparation of the skin surface was performed. A fresh pair of sterile gloves were worn to sample each participant, hands were washed, and hand sanitiser applied prior to changing gloves. The flocked swab was premoistened in DNA-free water (Thermofisher Scientific, Australia) in sterile 2mL cryovials. Each area was sampled with the flocked swab over the area pre-marked using the standardised stencil rubbing the swab in multiple directions over ten seconds.

Superficial skin scrapings were obtained by taking multiple strokes using the edge of a glass microscope slide from the area pre-marked using the standardised stencil over approximately ten seconds until there were visible stratum corneum (dead skin) cells present on the glass microscope slide. Visible scrapings on the glass microscope slide were then collected using a flocked swab pre-moistened in PCR grade water in sterile 2mL cryovials. For the control skin scraping, the slide package was opened, exposed to air, then a flocked swab pre-moistened in PCR grade water was wiped over the surface of the slide.

All collected swabs and scrape samples were stored in labelled 5mL sterile tubes containing PrimeStore® (Longhorn Vaccines and Diagnostics, Bethesda, Maryland, USA). Samples were stored at −80°C upon reaching the laboratory and until further processing.

### 2.4 DNA extraction, PCR, and sequencing

DNA was extracted from thawed swab and scrape samples in PrimeStore® using MagMax Ultra Nucleic acid kit (ThermoFisher Scientific, Australia) with a few modifications to the manufacturer’s protocol. Briefly, 500 µl of sample was placed in sterile 2 ml tubes, 40 µl of proteinase K and 260 µl of lysis buffer was added and the solution was heated at 60°C for 1 hour. Negative controls (PCR grade water containing no skin sample) and a positive control containing ZymoBIOMICS mock community (Zymo Research, USA) were processed in parallel. All samples were subject to bead beating (Qiagen Tissue Lyser, Qiagen, Germany) at highest frequency for 10 min after incubation. DNA was extracted following manufacturer’s instructions using the automated KingFisher Flex platform. All extracted DNA was eluted in 50 µl of elution buffer and stored at –20°C until further processing.

The full length 16S rRNA gene was amplified using the Pacific Biosciences^®^ (PacBio) protocol with 27F (GCAGTCGAACATGTACGCTGACTCAGGTCACAGRGTTYGATYMTGGCTCAG) and 1429R (GCAGTCGAACATGTACGCTGACTCAGGTCACRGYTACCTTGTTACGACTT) barcoded universal primers. The DNA was amplified and barcoded in a single PCR reaction using 8 forward and 24 reverse barcodes from PacBio (Procedure and Checklist Part number 101599500 V4). 25 µl reactions were set up using 12.5 µl of 2X KAPA HiFi HotStart ReadyMix (Roche, USA), 2.5 µM forward barcoded primer, 2.5 µM of reverse barcoded primer and 5 µl of extracted DNA. PCR reaction conditions were set for denaturation at 95°C for 3 minutes followed by 35 cycles of denaturation at 95°C for 30 sec, annealing at 57°C for 30 sec at a ramp rate of 2.5 and extension at 72°C for 60 sec. A final extension step of 72°C for 7 minutes was included. A negative control was included in all PCR runs using 5 ml of PCR grade water instead of DNA template.

Amplification of all samples was confirmed using QIAxcel Advanced Analyser (Qiagen, Germany), with a QIAxcel^®^ DNA High Resolution Kit (1200) (Qiagen, Germany). Amplicons were quantified using Qubit^®^ 2.0 Fluorometer with Qubit High sensitivity kit (ThermoFisher Scientific, USA). Finally, the samples were pooled in equimolar concentrations. The pool was cleaned using AMPure^®^ PB Bead (Beckman and Coulter, USA) kit as per manufacturers protocol. The final pool was tested for amplicon size by and quantified using Qubit^®^ 2.0 Fluorometer and QIAxcel Advanced Analyser. SMRTbell libraries were prepared according to Pacific Biosciences protocol. The prepared libraries were sequenced on PacBio Sequel II at Genomics WA, Perth, Australia.

### 2.5 Quantitative PCR

Quantitative PCR (qPCR) was carried out to quantify bacterial 16S rRNA gene copy number to estimate the total bacterial load of each sample. A reaction volume of 30 µl was prepared using 15 µl of TaqMan™ fast advance mastermix (Applied Biosystems, ThermoFisher Scientific, USA), 0.33 µM concentration of forward primer (5’-CGA AAG CGT GGG GAG CAA A-3’) and reverse primer (5’-GTT CGT ACT CCC CAG GCG G-3’). A final concentration of 0.33 µM probe was added (FAM-ATT AGA TAC CCT GGT AGT CCA-MGB) with 2.5 µl of DNA template and 9.5 µl of PCR grade water. A serially diluted standard of purified *Neisseria meningitidis* gDNA was included to estimate gene copy number. The qPCR reactions were performed using Applied Biosystems QuantStudio™ 6 Pro (ThermoFisher Scientific, USA) real time PCR instrument. The reaction plate was run for 40 cycles including an initial denaturation of 95°C for 120 sec, followed by denaturation at 95°C for 1 sec and annealing at 60°C for 20 sec (Bogaert et al., 2011). All amplification results were visualised using Design and Analysis software v2.6.0.

### 2.6 Bioinformatics and statistical analysis

Sequences were converted to circular consensus sequences (CCS) reads and demultiplexed using SMRT Link software V 11.1. CCS reads were filtered to retain only those with a minimum of three full passes and 99% sequence accuracy with a PHRED quality score of >30. Demultiplexed sequences were processed through an in-house Nextflow pipeline (https://github.com/PacificBiosciences/pb16nf/tree/b2d55b3464994c1026227d1624758c193d3c2b1e). Briefly, primers were removed, and sequences were denoised with min Q20 and max_EE set to 2 and chimeras removed using DADA2 (Callahan et al., 2016) pipeline. A feature table consisting of amplicon sequences variants (ASVs) was generated. ASVs were classified using Naïve-Bayes classifier function and the “besttax” function in the Nextflow pipeline. This function uses three databases, GTDB, Silva v138 and RefSeq+RDP sequentially to maximise ASV species level assignment.

The negative controls were used to remove potential contaminants from the assigned taxonomy feature table using Decontam package in R software (Davis et al., 2018). Gene copy number calculated from qPCR was used as the quantitation input. Contaminants were identified according to the statistical score using frequency and prevalence combined mode. All ASVs identified as contaminants were removed from the feature table. The resulting clean ASVs table was used for all the downstream analysis.

All univariate statistical analysis were performed in R software v4.2 using Vegan v2.5.7 (Oksanen et al., 2020) and Lme4 package (Bates et al., 2015). Alpha (α) diversity was calculated at ASVs level as Chao1 index and Shannon Weiner Index. Statistically significant groups were identified using linear mixed models (LMM) including random effects for participant to account for multiple samples per participant. LMM was estimated using REML and nloptwrap optimizer. The normality of the data was confirmed using Shapiro-Wilk test. For indices that were not normally distributed, we applied generalised linear models (gLM). Beta diversity (β) was analysed with nonmetric multidimensional scaling (NMDS) using Bray-Curtis dissimilarity index at ASVs level using Vegan and Phyloseq (McMurdie and Holmes, 2013) packages. Statistical significance was analysed using PERMANOVA in R in the Vegan package. All plots were produced using ggplot2 package in R (Wickham, 2011).

We also carried out differential abundance analysis using the MaAsLin2 package in R software (Mallick et al., 2021) including ‘participant’ as a random effect to account for multiple samples from the same participant. p-values derived from MaAsLin2 were adjusted using the Benjamin-Hochberg false discovery rate (FDR), and q-Values and coefficients were determined.

## 3 Results

Six healthy children (4 male, 2 female), aged 3 to 9 years, were included (**Table 1**) and all attended the follow up visit one week later (**Supplementary Figure 1**).

**Table 1:** Baseline characteristics of participants in study.

Characteristics	
Age years, mean (range)	6.5 (3–9)
Male (%)	4/6 (66.7%)
Ethnicity: Caucasian (%)	6/6 (100%)
Birth: Vaginal delivery (%)	6/6 (100%)
Breastfed (%)	6/6 (100%)
Length of breastfeeding, months (range)	7.5 (6–9)
Co-morbidities:	
Nose problems (occasional nose bleeds) (%)	1/6 (16%)
Skin problems (eczema, keratosis pilaris, port wine stain, molluscum) (%)	2/6 (33%)
Allergies (including food, drug, other) (%)	0 (0%)
Recent skin infection (molluscum)	1/6 (16%)
Other:	
Fingernail bitter (%)	5/6 (83%)
Family history of skin conditions (eczema, keratosis pilaris, skin cancer)	5/6 (83%)
Recent antibiotics (%)	1/6 (13%)
Regular sunscreen use (%)	6/6 (100%)
Regular use of topical antiseptics/antibacterials (%)	0/6 (0%)
Regular use of topical steroids (%)	1/6 (13%)
Daily frequency of bathing/showering (%)	6/6 (100%)
Use of probiotics/probiotic yoghurt (%)	2/6 (33%)
Swum in 7 days prior to sample collection (1^st^ visit)	5/6 (83%)
Swum in 7 days prior to sample collection (2^nd^ visit)	4/6 (66%)

All children were born via vaginal delivery, were previously breastfed, had no known allergies, and did not take any regular oral medication. All participants bathed daily, regularly used sunscreen and five participants had swum in the 7 days prior to samples being collected for the first visit and four participants had swum prior to the second visit. One child had recently received antibiotics (cephalexin, ceased two weeks prior to sampling) and one child used topical steroids regularly (methylprednisolone aceponate 0.1% ointment applied daily to the cubital fossa). However all children met the inclusion criteria and were instructed to avoid moisturisers, sunscreens, or creams on the child’s skin in the morning of areas being sampled, avoid swimming in a chlorinated pool or ocean in the 24 hours prior to sampling and were advised not to apply any topical antiseptics on the skin in the 48 hours prior to sampling.

In total 160 samples were collected, including 78 flocked swabs, 78 skin scrapings and four sampling controls. All swabs and skin scrapings were analysed including controls. Following quality filtering 1,678,456 reads were assigned to 6771 ASVs. These ASVs were successfully classified to 6137 genera and 5297 species. The Decontam pipeline identified 38 potential contaminants all of which were removed from the feature table (**Supplementary Table 1**).

### 3.1 Skin microbiome diversity and composition differs by body site

**Figure 1:**
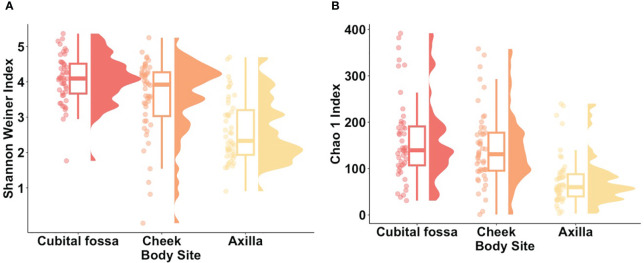
Alpha diversity metrics for each body site (cubital fossa, cheek, and axilla). **(A)** Raincloud plots of Shannon Weiner index (Cubital fossa; p value = < 0.001, b = 1.44, Cheek; p value = < 0.001, b = 0.93, comparator axilla) and **(B)** Raincloud plots of Chao 1 Index (Cubital fossa; p value = < 0.001, b = 1.44, Cheek; p value = <0.001, b = 68.47, comparator axilla) at amplicon sequence variant (ASV) level. The box plot represents the interquartile range, and the middle line represents the median. The coloured shape represents the density of diversity at each body site and the dots represent the distribution of individual participant samples.

Within-participant alpha (α-) diversity, as measured by Shannon Weiner index, differed between body sites. Cubital fossa (beta = 1.44, 95% CI [1.09, 1.79]) and cheek (beta = 0.93, 95% CI [0.58, 1.28]) had higher diversity than axilla (**Figure 1**). Similar findings were observed using a measure of richness (Chao 1), (**Figure 1**), with cubital fossa (beta = 85.80, 95% CI [57.82, 113.78]) and cheek (beta = 68.47, 95% CI [40.49, 96.45]) having higher richness than axillary samples.

**Figure 2:**
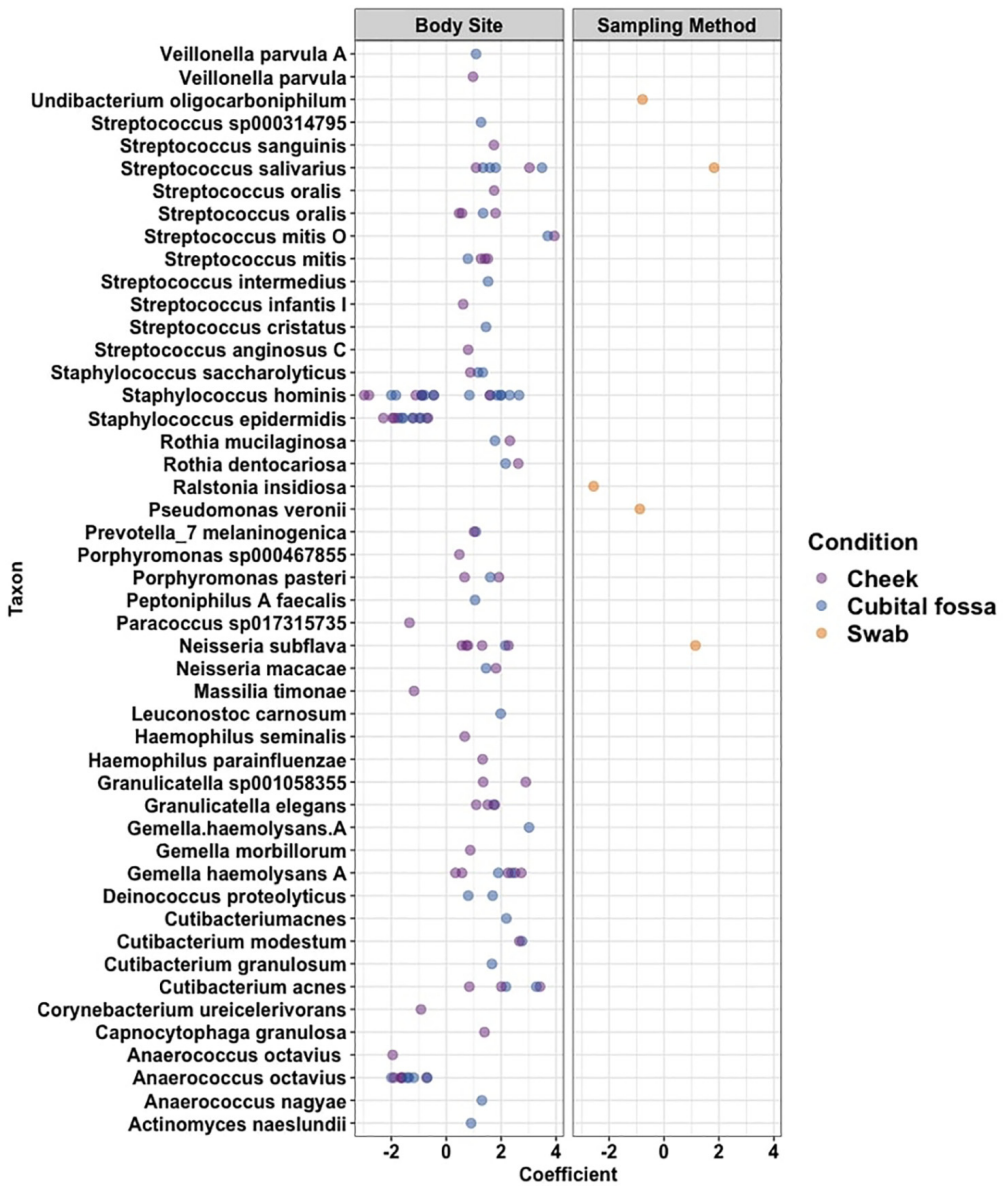
Cleveland plot showing differential abundance coefficients for species which were significantly different (i) between body sites (cubital fossa or cheek, with axilla as reference), and (ii) between sampling methods (flocked swab samples with scrape samples as reference) as determined using MaAslin2 analysis.

**Figure 3:**
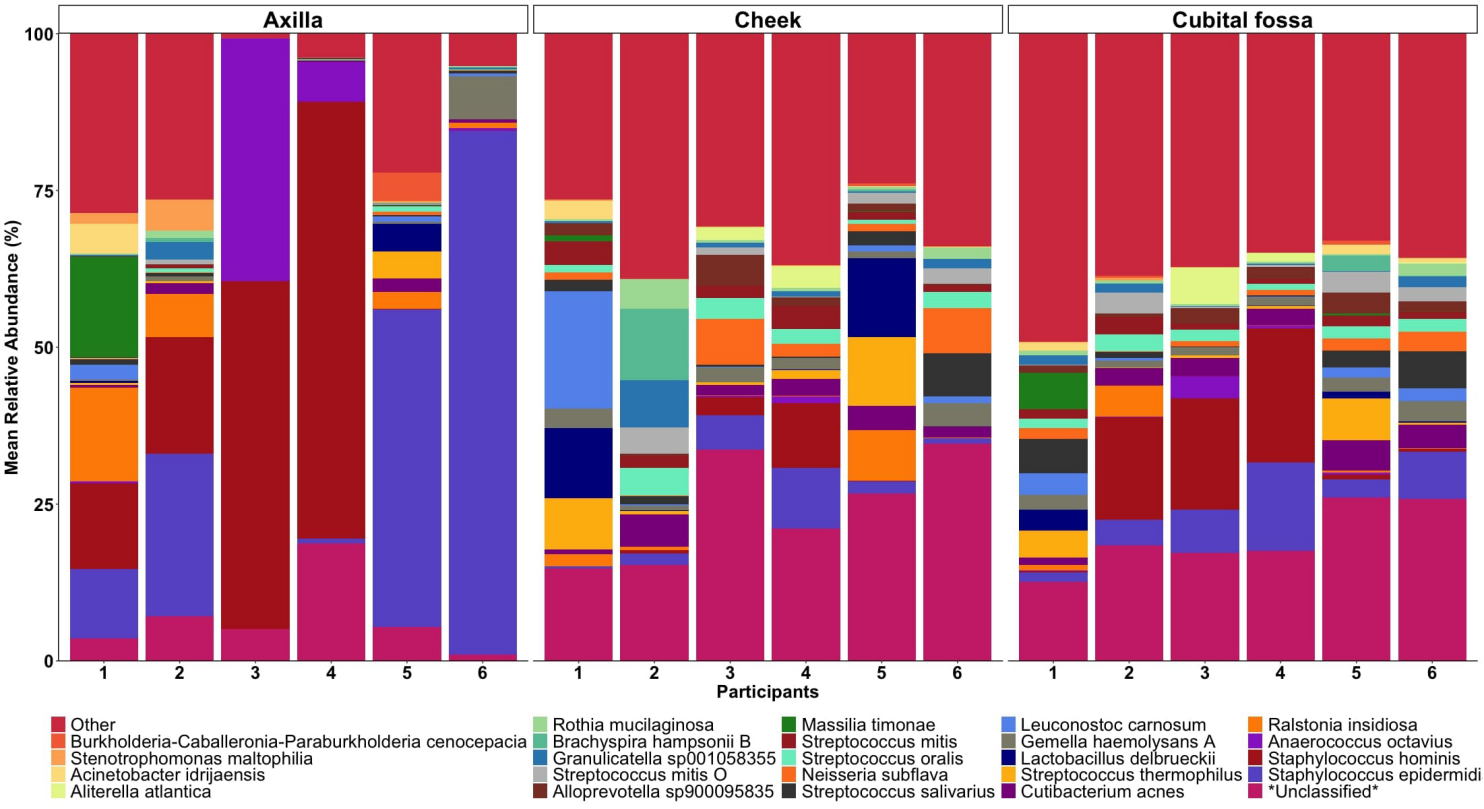
Mean relative abundance bar plot of 30 taxa (at the lowest taxonomic assignment) with the highest relative abundances, stratified by body site (axilla, cheek, and cubital fossa).

**Figure 4:**
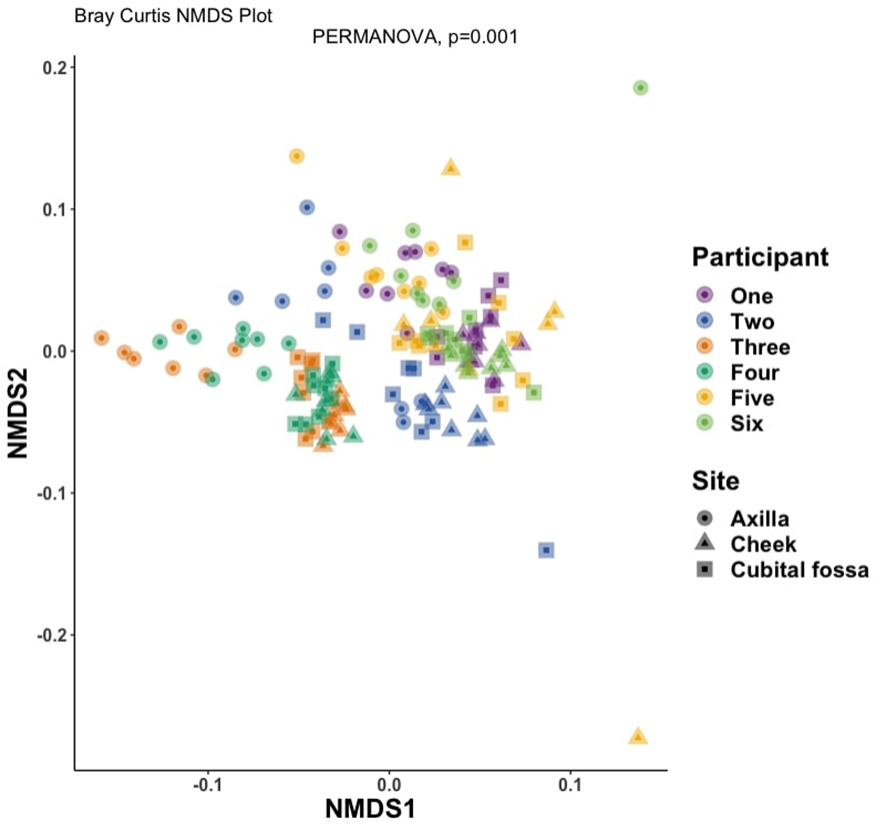
Non-metric multidimensional scaling (NMDS) plot showing distances between samples, coloured by participant number and with shapes indicating body site, at amplicon sequence variant (ASV) level using Bray-Curtis dissimilarity index (PERMANOVA for body site, p-value = 0.001).

Differential abundance analysis revealed 129 statistically significant species in cubital fossa and cheek when compared to axillae. Most notably, *Cutibacterium modestum* [coeff 2.75; qvalue 1.57E-05] and *Staphylococcus hominis* [coeff 2.30; qvalue 2.48E-05] were significantly higher in cubital fossa compared to axillae (**Figure 2**; **Supplementary Table 2**). *Streptococcus mitis* had increased relative abundance in cheek samples [coeff 1.51; qvalue 7.41E-07] whereas, *Staphylococcus hominis* had lower relative abundance in cheek samples compared to axilla [coeff −2.98; qvalue 1.38E-05]. The bar plot (**Figure 3**) shows the bacterial composition at different body sites. Axillary samples showed a higher relative abundance of *Staphylococcus hominis* and *Staphylococcus epidermidis.* Beta-diversity was visualised using an NMDS plot, and showed clustering by participant and by body site (Sampling method; PERMANOVA p = 0.01, Body site; PERMANOVA p= 0.001) (**Figure 4**). Finally, we also analysed the left and right side of the body which showed no significant differences between the body sides (**Supplementary Figure 2**).

### 3.2 Sampling method has relatively little impact on microbial composition and the skin microbiome is stable longitudinally

**Figure 5:**
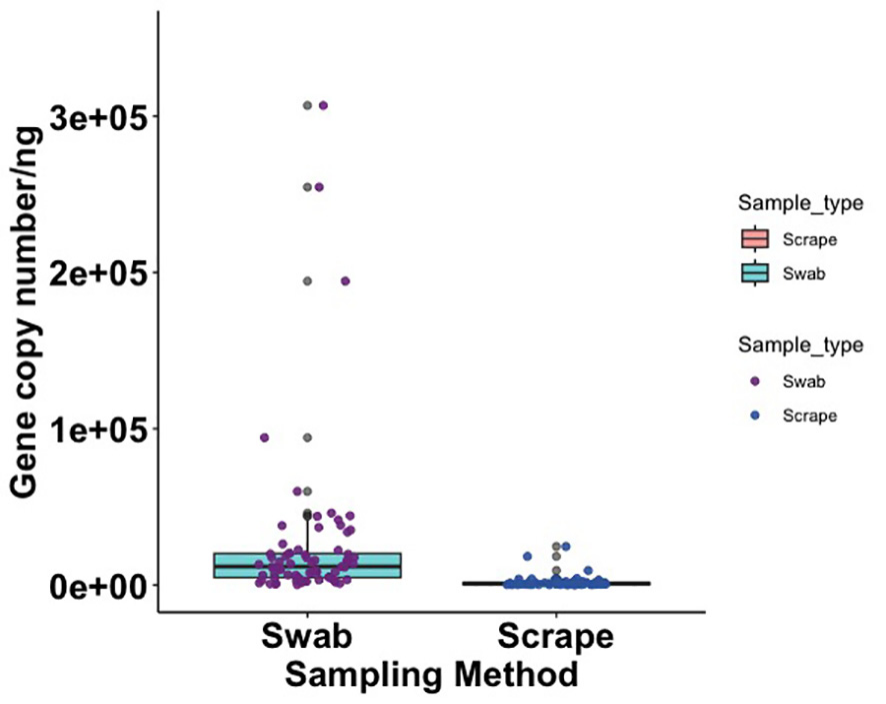
Box plot showing 16S rRNA gene copy number per nanogram of extracted DNA from flocked swab and scrape samples (Kruskal Wallis, p = < 0.001).

**Figure 6:**
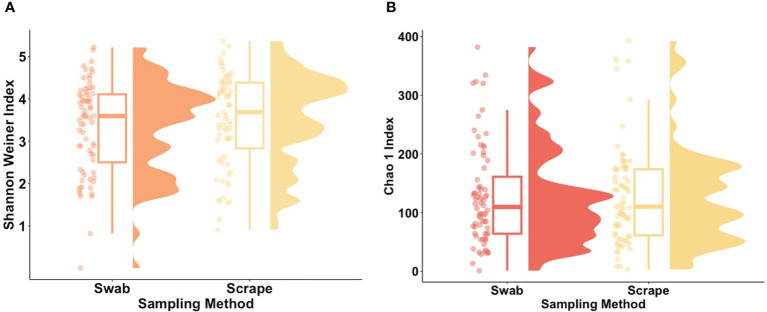
Alpha diversity metrics for flocked swab and scrape samples. **(A)** Raincloud plot of Shannon Weiner index (Swab; p value = 0.31, b = −0.18) and **(B)** Raincloud plot of Chao 1 Index (Swab; p value = 0.31, b = −0.74) at amplicon sequence variant (ASV) level. The box plot represents the interquartile range, and the middle line represents the median. The coloured shape represents the density of diversity at each sampling method and the dots represent the distribution of individual participants.

**Figure 7:**
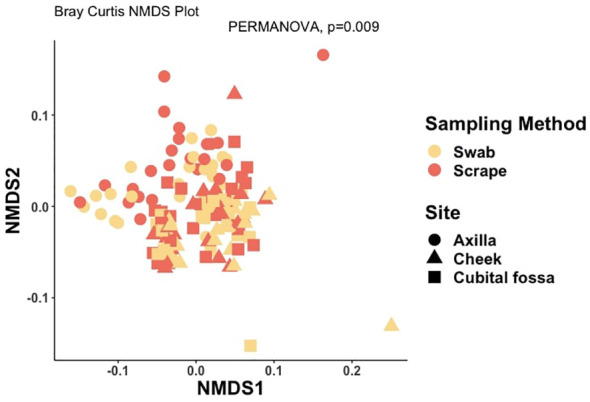
Non-metric multidimensional scaling (NMDS) plot showing distances between samples, coloured by sample type and with shapes indicating body site, at amplicon sequence variant (ASV) level using Bray-Curtis dissimilarity index (PERMANOVA for sampling method, p-value = 0.009).

The bacterial load (qPCR for 16S rRNA gene) of flocked swab samples was significantly higher than that of scrapings (**Figure 5**) (Kruskal-Wallis, p <0.001). Alpha diversity was not significantly different between sampling methods (Shannon Weiner index: beta = −0.18, 95% CI [−0.53, 0.17]; Chao1: beta = −0.74, 95% CI [−26.67, 25.20]), (**Figure 6**). On differential abundance analysis (**Figure 2**) only *Ralstonia insidiosa* species was showed a significant difference by sample type, and was negatively associated with swab samples [coeff −2.54, qvalue 0.001]. To explore differences in overall bacterial diversity by sample type, Bray Curtis dissimilarity index was calculated and showed higher between-sample type than within sample-type distances (PERMANOVA, p=0.009), although samples largely overlapped on NMDS plots (**Figure 7**).

**Figure 8:**
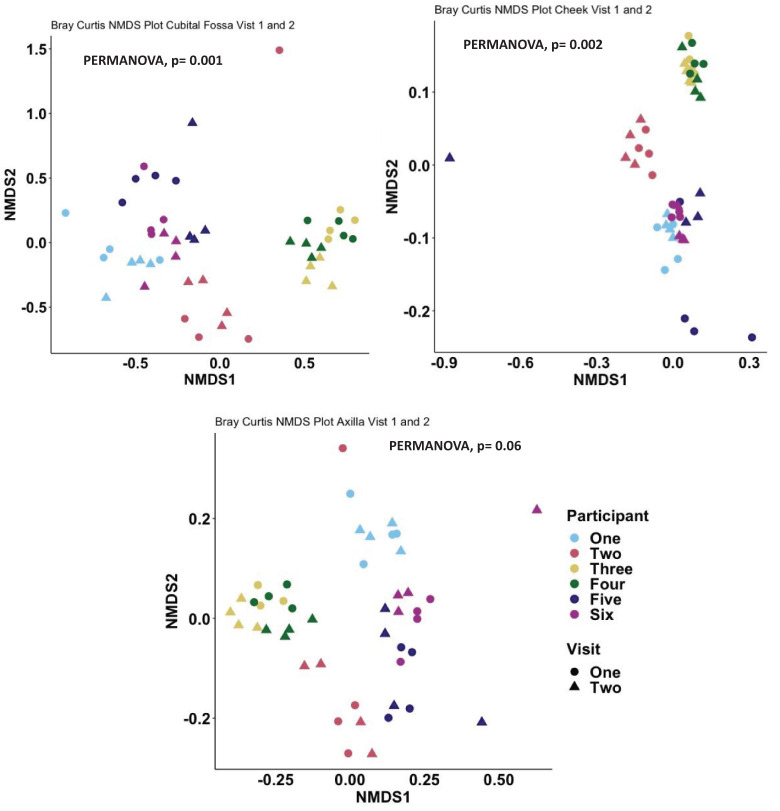
Non-metric multidimensional scaling (NMDS) plot showing distances between samples, coloured by participant and with shapes indicating visit number, at amplicon sequence variant (ASV) level using Bray-Curtis dissimilarity index, Cubital fossa (PERMANOVA, p-value = 0.001), Cheek (PERMANOVA, p-value = 0.002) and Axilla (PERMANOVA, p-value 0.06).

**Figure 9:**
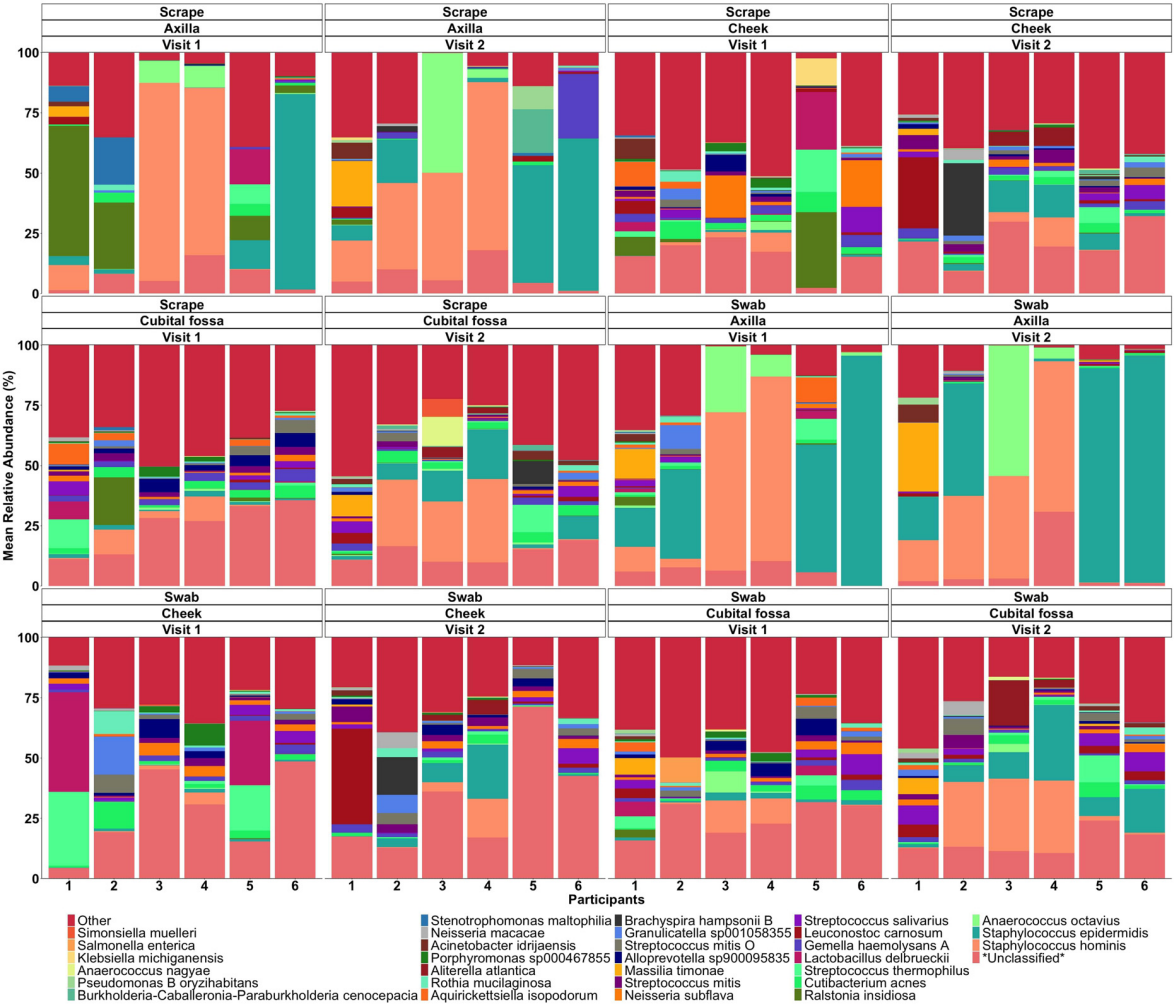
Mean relative abundance bar plot of top species for scraping and swab samples of each participant at different body sites and sampling visits.

When comparing the overall bacterial composition within participants and sites across the two visits, the composition of the microbiome appeared largely stable over time, with samples from the same site and participants clustering closely between visits. However, on statistical analysis, there were significant differences over time for cubital fossa (PERMANOVA, p=0.001) and cheek (PERMANOVA, p=0.01), but not for axilla (PERMANOVA, p=0.06) (**Figure 8**). However the plots demonstrate that between participant differences are much larger than within participant differences over time and that differences over time vary between individuals, with samples from participant five showing more variability over time than other participants. The skin microbiome diversity was significantly different over a time of one week for all participants and can be seen in the composition plot (**Figure 9**).

## 4 Discussion

In this study, we compared flocked swabs and skin scrapings for analysis of the skin microbiome using full length 16S rRNA gene sequencing. There is currently no standardised protocol for skin sampling for microbiome studies. Our study contributes to this gap by identifying and optimising a preferred sampling strategy. Overall, our results support flocked swabs as the preferred sampling method (over skin scrapings) for sampling the skin microbiome in children. Microbial composition did not differ substantially with sample type, however less microbial DNA was collected using the scraping method, leading to a higher risk of contaminants being over-represented. Although no formal assessment was made, we also considered the participant comfort, safety, and tolerability in children, when recommending swabbing over scraping. The flocked swab sampling technique has additional benefits as it requires less steps and fewer consumables. We also believe it would be better tolerated by adults and children, including neonates. The majority of dissimilarity between the samples in this study was accounted for by differences between the participants and differences between the sampling sites. Recent studies used flocked swabs in extremely preterm infants during the first three weeks of life (Ghori et al., 2023). This study reported on dissimilarity at different body sites and found between-body-site distances were greater than within-site distances.

There was no significant difference in microbial diversity and richness between the two sampling methodologies, with only minor differences in composition involving less abundant taxa. The similarity of results allowed us to consider preferable sampling based on other factors e.g., participant tolerability. In this pilot study, data was not collected in advance on patient tolerability as there was not expected to be a difference. However, informal responses from all participants were that the flocked swab was less irritating and preferred. This extends the finding from Bjerre et al. who reported a very large overlap in operational taxonomic unit (OTU) identified and OTU counts when comparing eSwabs and skin scrapes (Bjerre et al., 2019). Grice et al. (2008), reported that with three methods of sampling (swab, scrape, and punch biopsy), at all depths of sampling Proteobacteria dominated the skin microbiota (Grice et al., 2008). As such it has been postulated that the microbiota present on scrapes and swabs may be representative of skin differentiation history. The external microbiome, whether it be alive or dead, may reflect the physiological roles and processes of the microbiome deeper within the skin (Bjerre et al., 2019; Grice et al., 2008; Nakatsuji et al., 2013). This study and previous studies did not differentiate between dead and viable cells. However, Nakatsuji et al. (2013) previously reported that microorganisms do not have to be alive to influence the host immune system (Nakatsuji et al., 2013).

In sampling the skin microbiome of three body sites namely, cubital fossa, cheek and axilla our results showed that there was marked interpersonal variability, with each body site showing different taxa for each participant. *Streptococcus mitis* had higher relative abundance in cheek samples [coeff 1.51; qvalue 7.41E-07] whereas *Staphylococcus hominis* lower relative abundance in cheek samples, compared with axillary samples [coeff −2.98; qvalue 1.38E-05]. The bar plot (**Figure 3**) shows the composition of taxonomy at different body sites. Meisel et al. (2016) sampled skin sites across a range of microenvironments including moist sites (umbilicus and toe web space), sites that were intermittently moist (palm and antecubital fossa) as well as sebaceous (forehead, occiput, retro auricular crease) (Meisel et al., 2016). Whilst Meisel et al. (2016) did not sample the axilla, the study reported that independent of method, moist sites were taxonomically most comparable across all sequencing methods (using two widely sequenced regions of the 16S rRNA gene and whole metagenome shotgun sequencing). We found that axillary samples had a more stable microbiome over a short period of time, compared with other body sites. Whether this is also true for adults with changes around puberty due to hormone related changes, who are more likely to apply anti-perspirants and other products as well as have varying hair removal practices deserves investigation.

The microbiome between the left and right sides of the same body were similar in the same participant, suggesting that sampling only one site is sufficient to capture site-related differences in microbial composition. In addition, in our study one operator sampled the left side and another operator sampled the right side suggesting that our sampling methodology was consistent and reproducible between operators. This finding should also be considered when investigating microbial changes in the context of asymmetrical skin disease or trauma when sampling both the involved and the contralateral body site may be informative. The microbiome was relatively stable over longitudinal sampling, although this varied between participants.

This study was a small study population (six participants) and a homogenous population (healthy Caucasian children, metropolitan site), and few body sites sampled. The aim of our study was to compare sampling methods, not to capture diversity within a population. Even within this homogenous group of children there was a significant difference in the ‘normal’ skin microbiome, and as such, very large studies will likely be needed to define the normal skin microbiome in groups with different ages, races, diet, lifestyle, and location (e.g., rural vs urban).

Our study provides unique methodological insights into sampling, as one of a small number of skin microbiome studies reported to date in children. Since we employed compositional methods, we are not able to infer absolute abundance of taxa. We only sampled three body sites. Whilst these are broadly representative of different skin types, these are not fully representative of the surface of the largest organ of the body. We used multiple controls to detect potential contamination and corrected for potential contaminants, however, all amplicon-based methods for sampling low-biomass communities may be affected by contamination.

It is difficult to compare our results to the results and conclusions of previous skin microbiome studies due to the biases in skin microbiome sample preparation and analysis (Santiago-Rodriguez et al., 2023). For example, in the process of recovering DNA from samples in skin microbiome studies, variation can be introduced, or gram-negative bacteria are sometimes over-represented (Bjerre et al., 2019). In addition, due to the low biomass of the skin microbiome, extraction needs to reduce the external bioburden (otherwise known as the “kitome”) that may confound the interpretation of the true microbiome of the sample (Bjerre et al., 2019). However, overall, the predominant taxa which we identified in this study were similar to those found in other studies (Meisel et al., 2016; Byrd et al., 2018).

This study allows future novel work exploring the skin microbiome in clearly defined infectious diseases such as impetigo, in skin diseases that are exacerbated by infection e.g., eczema, in the process of skin healing following a burn injury and in skin diseases that are treated with long term antibiotics for a presumed but currently poorly defined role of bacterial pathogenesis e.g., acne. Understanding normal skin flora will help define how microbial imbalance may be associated with skin disease and skin healing. This will be invaluable in populations with a high burden of skin disease (e.g., children living in remote Indigenous communities in Australia who have the highest reported rates of impetigo in the world (Bowen et al., 2015). Future studies could also have wider implications for health in terms of skin-gut microbiome axis and impact on systemic infection and disease states (Piewngam et al., 2023).

## Data Availability

The data presented in the study are deposited in the NCBI repository, accession number PRJNA1168673.
