# Supplementary material for: Optimisation of the sampling method for skin microbiome studies in healthy children: a pilot cohort study
Source: Front Microbiomes. 2024 Nov 12;3:1446394. doi: 10.3389/frmbi.2024.1446394 (PMC12993633; doi:10.3389/frmbi.2024.1446394)
Supplement: Supplementary file 1 [file DataSheet1.docx]

**Supplementary material**

**Table 1**

| **ASV number** | **Taxonomy** |
| --- | --- |
| ASV 4 | d__Bacteria; p__Proteobacteria; c__Gammaproteobacteria; o__Pseudomonadales; f__Pseudomonadaceae; g__Pseudomonas_E; s__Unclassified |
| ASV5 | d__Bacteria; p__Proteobacteria; c__Gammaproteobacteria; o__Pseudomonadales; f__Pseudomonadaceae; g__Pseudomonas_E; s__Unclassified |
| ASV9 | d__Bacteria; p__Proteobacteria; c__Gammaproteobacteria; o__Burkholderiales; f__Burkholderiaceae; g__Ralstonia; s__Ralstonia insidiosa |
| ASV77 | d__Bacteria; p__Proteobacteria; c__Gammaproteobacteria; o__Burkholderiales; f__Comamonadaceae; g__Variovorax; s__Variovorax paradoxus |
| ASV115 | d__Bacteria; p__Proteobacteria; c__Gammaproteobacteria; o__Burkholderiales; f__Burkholderiaceae; g__Comamonas; s__Comamonas sp002754475 |
| ASV152 | d__Bacteria; p__Actinobacteria; c__Actinobacteria; o__Micrococcales; f__Micrococcaceae; g__Arthrobacter; s__Arthrobacter russicus |
| ASV167 | d__Bacteria; p__Proteobacteria; c__Gammaproteobacteria; o__Pseudomonadales; f__Pseudomonadaceae; g__Pseudomonas_E; s__Unclassified |
| ASV233 | d__Bacteria; p__Bacteroidota; c__Bacteroidia; o__Flavobacteriales; f__Weeksellaceae; g__Chryseobacterium; s__Chryseobacterium indologenes |
| ASV263 | d__Bacteria; p__Proteobacteria; c__Alphaproteobacteria; o__Rhizobiales; f__Rhizobiaceae; g__Rhizobium; s__Unclassified |
| ASV327 | d__Bacteria; p__Firmicutes; c__Bacilli; o__Staphylococcales; f__Staphylococcaceae; g__Staphylococcus; s__Staphylococcus hominis |
| ASV380 | d__Bacteria; p__Proteobacteria; c__Gammaproteobacteria; o__Burkholderiales; f__Burkholderiaceae; g__Herbaspirillum; s__Herbaspirillum sp013426985 |
| ASV403 | d__Bacteria; p__Proteobacteria; c__Gammaproteobacteria; o__Burkholderiales; f__Oxalobacteraceae; g__Herbaspirillum; s__Herbaspirillum huttiense |
| ASV492 | d__Archaea; p__Asgardarchaeota; c__Lokiarchaeia; o__CR-4; f__SOKP01; g__SOKP01; s__SOKP01 sp013375475 |
| ASV556 | d__Bacteria; p__Proteobacteria; c__Gammaproteobacteria; o__Enterobacterales; f__Pasteurellaceae; g__Haemophilus_A; s__Haemophilus A parahaemolyticus |
| ASV567 | d__Bacteria; p__Proteobacteria; c__Alphaproteobacteria; o__Rhizobiales_A; f__Rhizobiaceae_A; g__Phyllobacterium; s__Phyllobacterium myrsinacearum |
| ASV749 | d__Bacteria; p__Proteobacteria; c__Alphaproteobacteria; o__Sphingomonadales; f__Sphingomonadaceae; g__Sphingomonas; s__Sphingomonas daechungensis |
| ASV886 | d__Bacteria; p__Deinococcus-Thermus; c__Deinococci; o__Deinococcales; f__Deinococcaceae; g__Deinococcus; s__Deinococcus aetherius |
| ASV896 | d__Bacteria; p__Firmicutes; c__Bacilli; o__Staphylococcales; f__Staphylococcaceae; g__Staphylococcus; s__Staphylococcus haemolyticus |
| ASV949 | d__Bacteria; p__Proteobacteria; c__Alphaproteobacteria; o__Rhodobacterales; f__Rhodobacteraceae; g__Paracoccus; s__Unclassified |
| ASV1127 | d__Bacteria; p__Firmicutes_A; c__Clostridia; o__Tissierellales; f__Peptoniphilaceae; g__Parvimonas; s__Parvimonas sp000214475 |
| ASV1154 | d__Bacteria; p__Proteobacteria; c__Alphaproteobacteria; o__Caulobacterales; f__Caulobacteraceae; g__Brevundimonas; s__Brevundimonas vesicularis |
| ASV1224 | d__Bacteria; p__Actinobacteriota; c__Actinobacteria; o__Corynebacteriales; f__Corynebacteriaceae; g__Corynebacterium; s__Corynebacterium tuberculostearicum |
| ASV1237 | d__Bacteria; p__Actinobacteriota; c__Actinomycetia; o__Mycobacteriales; f__Mycobacteriaceae; g__Rhodococcus; s__Rhodococcus qingshengii |
| ASV1252 | d__Bacteria; p__Proteobacteria; c__Alphaproteobacteria; o__Sphingomonadales; f__Sphingomonadaceae; g__Sphingomonas; s__Sphingomonas olei |
| ASV1328 | d__Bacteria; p__Actinobacteriota; c__Actinomycetia; o__Actinomycetales; f__Micrococcaceae; g__Glutamicibacter; s__Unclassified |
| ASV1433 | d__Bacteria; p__Cyanobacteria; c__Cyanobacteriia; o__Cyanobacteriales; f__Unclassified; g__Unclassified; s__Unclassified |
| ASV1538 | d__Bacteria; p__Firmicutes_A; c__Clostridia; o__Tissierellales; f__Peptoniphilaceae; g__Anaerococcus; s__Anaerococcus prevotii |
| ASV1658 | d__Bacteria; p__Proteobacteria; c__Alphaproteobacteria; o__Sphingomonadales; f__Sphingomonadaceae; g__Sphingomonas; s__Sphingomonas turrisvirgatae |
| ASV1699 | d__Bacteria; p__Firmicutes; c__Clostridia; o__Clostridiales; f__Lachnospiraceae; g__Lachnoanaerobaculum; s__Lachnoanaerobaculum gingivalis |
| ASV1701 | d__Bacteria; p__Proteobacteria; c__Gammaproteobacteria; o__Xanthomonadales; f__Xanthomonadaceae; g__Stenotrophomonas; s__Stenotrophomonas tumulicola |
| ASV1736 | d__Bacteria; p__Bacteroidota; c__Bacteroidia; o__Flavobacteriales; f__Flavobacteriaceae; g__Capnocytophaga; s__Capnocytophaga ochracea |
| ASV1863 | d__Bacteria; p__Bacteroidota; c__Bacteroidia; o__Flavobacteriales; f__Weeksellaceae; g__Chryseobacterium; s__Chryseobacterium indologenes |
| ASV1883 | d__Bacteria; p__Proteobacteria; c__Gammaproteobacteria; o__Burkholderiales; f__Burkholderiaceae; g__Hylemonella; s__Hylemonella delicata |
| ASV2004 | d__Bacteria; p__Proteobacteria; c__Alphaproteobacteria; o__Rhodobacterales; f__Rhodobacteraceae; g__Paracoccus; s__Paracoccus sanguinis |
| ASV2477 | d__Bacteria; p__Firmicutes_A; c__Clostridia; o__Peptostreptococcales; f__Peptostreptococcaceae; g__Peptostreptococcus; s__Peptostreptococcus canis |
| ASV3095 | d__Bacteria; p__Firmicutes_A; c__Clostridia; o__Lachnospirales; f__Lachnospiraceae; g__Johnsonella; s__Johnsonella sp900766185 |
| ASV3620 | d__Bacteria; p__Firmicutes; c__Bacilli; o__Bacillales; f__Bacillaceae; g__Bacillus; s__Bacillus anthracis |
| ASV3900 | d__Bacteria; p__Actinobacteriota; c__Actinomycetia; o__Mycobacteriales; f__Mycobacteriaceae; g__Corynebacterium; s__Corynebacterium sp900618065 |

**Table 2**

| **Taxon** | **condition** | **coefficient** | **qvalue** |
| --- | --- | --- | --- |
| Anaerococcus octavius | Cubital fossa | -1.9934804 | 0.00010501 |
| Gemella haemolysans.A | Cubital fossa | 3.01465943 | 0.0001078 |
| Staphylococcus hominis | Cubital fossa | 2.00302159 | 0.0001078 |
| Staphylococcus hominis | Cubital fossa | 1.59241808 | 0.0001078 |
| Neisseria subflava | Cubital fossa | 2.15219679 | 0.00012481 |
| Anaerococcus octavius | Cheek | -1.9521557 | 0.00012608 |
| Neisseria subflava | Cheek | 1.30540332 | 0.00012934 |
| Cutibacterium acnes | Cheek | 3.41419016 | 0.00017106 |
| Anaerococcus octavius | Cheek | -1.6364443 | 0.00017106 |
| Gemella haemolysans A | Cheek | 2.50284039 | 0.00017106 |
| Deinococcus proteolyticus | Cubital fossa | 1.68830099 | 0.0002262 |
| Streptococcus mitis O | Cheek | 3.9368988 | 0.0002352 |
| Anaerococcus octavius | Cheek | -1.8980741 | 0.0002352 |
| Streptococcus salivarius | Cheek | 3.0301581 | 0.00031806 |
| Rothia mucilaginosa | Cheek | 2.31754379 | 0.00031806 |
| Cutibacterium acnes | Cubital fossa | 3.28192681 | 0.00031806 |
| Gemella haemolysans A | Cheek | 2.73387969 | 0.00047287 |
| Streptococcus oralis | Cheek | 1.79451595 | 0.00048655 |
| Streptococcus mitis O | Cubital fossa | 3.69555524 | 0.00063741 |
| Staphylococcus epidermidis | Cheek | -1.8851839 | 0.00075494 |
| Gemella haemolysans A | Cubital fossa | 2.37548396 | 0.00079969 |
| Neisseria macacae | Cheek | 1.81178574 | 0.00091407 |
| Streptococcus salivarius | Cubital fossa | 1.33936407 | 0.00113262 |
| Staphylococcus saccharolyticus | Cubital fossa | 1.15860342 | 0.00124657 |
| Anaerococcus octavius | Cheek | -1.607769 | 0.00130416 |
| Rothia dentocariosa | Cheek | 2.62112423 | 0.00132275 |
| Ralstonia insidiosa | Swab | -2.5664068 | 0.00136118 |
| Staphylococcus epidermidis | Cheek | -2.2885046 | 0.00150058 |
| Anaerococcus octavius | Cheek | -1.6283138 | 0.00167894 |
| Gemella haemolysans A | Cheek | 2.25185007 | 0.00167894 |
| Staphylococcus epidermidis | Cubital fossa | -1.5611599 | 0.00168739 |
| Streptococcus salivarius | Cubital fossa | 1.79735188 | 0.00170618 |
| Granulicatella elegans | Cubital fossa | 1.72401653 | 0.00209686 |
| Streptococcus cristatus | Cubital fossa | 1.44778957 | 0.00214484 |
| Streptococcus mitis | Cheek | 1.41229417 | 0.00228995 |
| Streptococcus sp000314795 | Cubital fossa | 1.26710563 | 0.00228995 |
| Staphylococcus hominis | Cheek | 1.59328071 | 0.00261173 |
| Staphylococcus hominis | Cheek | -0.8802476 | 0.00261173 |
| Streptococcus intermedius | Cubital fossa | 1.52292517 | 0.00261173 |
| Porphyromonas pasteri | Cheek | 1.91706757 | 0.00295562 |
| Corynebacterium ureicelerivorans | Cheek | -0.9212705 | 0.00296185 |
| Staphylococcus hominis | Cubital fossa | -1.9959474 | 0.00296185 |
| Staphylococcus saccharolyticus | Cubital fossa | 1.33067266 | 0.00338632 |
| Staphylococcus hominis | Cheek | -0.4567417 | 0.00359861 |
| Staphylococcus hominis | Cubital fossa | -0.4567417 | 0.00359861 |
| Staphylococcus hominis | Cubital fossa | 1.85123896 | 0.00368968 |
| Gemella morbillorum | Cheek | 0.87309342 | 0.00452963 |
| Staphylococcus hominis | Cubital fossa | 2.65241915 | 0.00452963 |
| Haemophilus parainfluenzae | Cheek | 1.3238996 | 0.00475078 |
| Gemella haemolysans A | Cheek | 0.33602921 | 0.00496244 |
| Staphylococcus epidermidis | Cubital fossa | -0.7140941 | 0.00510715 |
| Anaerococcus octavius | Cubital fossa | -1.4167082 | 0.00560331 |
| Staphylococcus epidermidis | Cheek | -1.2152534 | 0.00615909 |
| Staphylococcus epidermidis | Cubital fossa | -1.2152534 | 0.00615909 |
| Cutibacterium granulosum | Cubital fossa | 1.66334019 | 0.00643822 |
| Capnocytophaga granulosa | Cheek | 1.3909193 | 0.00648422 |
| Streptococcus salivarius | Cubital fossa | 1.59056615 | 0.00648422 |
| Gemella haemolysans A | Cubital fossa | 1.8962744 | 0.00651544 |
| Cutibacterium acnes | Cubital fossa | 2.17245693 | 0.00661656 |
| Staphylococcus epidermidis | Cheek | -1.7532352 | 0.00681025 |
| Deinococcus proteolyticus | Cubital fossa | 0.80037984 | 0.00698975 |
| Pseudomonas veronii | Swab | -0.880636 | 0.00698975 |
| Streptococcus anginosus C | Cheek | 0.7980061 | 0.00722214 |
| Anaerococcus nagyae | Cubital fossa | 1.29680728 | 0.00747596 |
| Staphylococcus epidermidis | Cheek | -0.9651853 | 0.00756177 |
| Cutibacterium acnes | Cheek | 0.84044321 | 0.00756177 |
| Prevotella_7 melaninogenica | Cubital fossa | 1.07928619 | 0.00756177 |
| Granulicatella elegans | Cheek | 1.50951651 | 0.0081903 |
| Rothia mucilaginosa | Cubital fossa | 1.77526323 | 0.0081903 |
| Staphylococcus hominis | Cubital fossa | -0.7850424 | 0.00829971 |
| Undibacterium oligocarboniphilum | Swab | -0.7830409 | 0.00835106 |
| Staphylococcus epidermidis | Cheek | -0.6700184 | 0.00897455 |
| Veillonella parvula A | Cubital fossa | 1.08500366 | 0.00897455 |
| Massilia timonae | Cheek | -1.1741564 | 0.00910412 |
| Staphylococcus epidermidis | Cubital fossa | -0.944352 | 0.00910412 |
| Granulicatella sp001058355 | Cheek | 1.34437261 | 0.00931922 |
| Granulicatella elegans | Cheek | 1.09213165 | 0.00947359 |
| Staphylococcus hominis | Cubital fossa | -1.8307734 | 0.00947359 |
| Anaerococcus octavius | Cubital fossa | -1.1819876 | 0.0095214 |
| Rothia dentocariosa | Cubital fossa | 2.16078046 | 0.0097112 |
| Anaerococcus octavius | Cubital fossa | -1.3666571 | 0.00979727 |
| Neisseria macacae | Cubital fossa | 1.45055901 | 0.00993652 |
| Streptococcus salivarius | Cheek | 1.08382628 | 0.01017495 |
| Streptococcus salivarius | Swab | 1.82237317 | 0.01092561 |
| Streptococcus oralis | Cheek | 1.7442601 | 0.01133286 |
| Streptococcus sanguinis | Cheek | 1.73560104 | 0.01133286 |
| Leuconostoc carnosum | Cubital fossa | 1.98280277 | 0.01133286 |
| Peptoniphilus A faecalis | Cubital fossa | 1.04601734 | 0.01133286 |
| Paracoccus sp017315735 | Cheek | -1.3396508 | 0.01175831 |
| Streptococcus oralis | Cubital fossa | 1.34198478 | 0.01175831 |
| Neisseria subflava | Cheek | 0.78740626 | 0.01190384 |
| Streptococcus mitis | Cubital fossa | 0.78765499 | 0.01190384 |
| Cutibacterium acnes | Cheek | 2.00454146 | 0.01192155 |
| Neisseria subflava | Cheek | 0.56984969 | 0.01192155 |
| Staphylococcus epidermidis | Cubital fossa | -1.6234314 | 0.01192155 |
| Granulicatella elegans | Cheek | 1.76573192 | 0.01229845 |
| Staphylococcus hominis | Cheek | -1.1096939 | 0.0125845 |
| Neisseria subflava | Cheek | 0.72932838 | 0.01336265 |
| Porphyromonas pasteri | Cubital fossa | 1.60242864 | 0.01419559 |
| Anaerococcus octavius | Cheek | -0.7159362 | 0.01443861 |
| Streptococcus oralis | Cheek | 0.47011941 | 0.01460368 |
| Cutibacteriumacnes | Cubital fossa | 2.18931637 | 0.01460368 |
| Streptococcus infantis I | Cheek | 0.61489986 | 0.01574575 |
| Actinomyces naeslundii | Cubital fossa | 0.90407303 | 0.01685852 |
| Prevotella_7 melaninogenica | Cheek | 1.01143288 | 0.01771735 |
| Neisseria subflava | Swab | 1.14376023 | 0.01771735 |
| Staphylococcus saccharolyticus | Cheek | 0.87466967 | 0.01842505 |
| Streptococcus oralis | Cheek | 0.57298429 | 0.01842505 |
| Haemophilus seminalis | Cheek | 0.67198132 | 0.01842505 |
| Anaerococcus octavius | Cubital fossa | -0.6951029 | 0.01857662 |
| Staphylococcus hominis | Cheek | -0.8852928 | 0.01884959 |
| Gemella haemolysans A | Cheek | 0.57480278 | 0.01884959 |
| Staphylococcus hominis | Cubital fossa | -0.8852928 | 0.01884959 |
| Staphylococcus hominis | Cubital fossa | 0.84191569 | 0.01884959 |
| Porphyromonas pasteri | Cheek | 0.66645144 | 0.01960034 |
| Porphyromonas sp000467855 | Cheek | 0.47773023 | 0.01960034 |
| Veillonella parvula | Cheek | 0.97405191 | 0.01986028 |
| Staphylococcus hominis | Cheek | -2.9853317 | 1.38581422786792e-05 |
| Cutibacterium modestum | Cubital fossa | 2.75875996 | 1.57092925726766e-05 |
| Staphylococcus hominis | Cheek | -2.8153323 | 2.0719829399357e-05 |
| Staphylococcus hominis | Cubital fossa | 2.3063214 | 2.48527224515833e-05 |
| Cutibacterium modestum | Cheek | 2.66649313 | 2.5928716171224e-05 |
| Granulicatella sp001058355 | Cheek | 2.89769938 | 3.0573699906018e-05 |
| Streptococcus salivarius | Cubital fossa | 3.48571541 | 3.72848842508693e-05 |
| Staphylococcus hominis | Cubital fossa | 1.97965367 | 5.0913152912497e-05 |
| Streptococcus mitis | Cheek | 1.2651344 | 5.16959870830855e-05 |
| Neisseria subflava | Cheek | 2.26412591 | 5.80660773144845e-05 |
| Staphylococcus epidermidis | Cheek | -1.9501797 | 6.34895277203249e-05 |
| Streptococcus mitis | Cheek | 1.51303684 | 7.41949473891121e-07 |

**Supplementary Figure 1: Flow diagram for study participants**

All participants complete second round of sampling 1 week later

N = 6 (no participants lost to follow up)

All participants complete first round of sampling

N = 6

Enrolled participants

N = 6

Eligible participants

N = 6

Parents of all participants complete demographics questionnaire

N = 6

**Supplementary Figure 2**

**
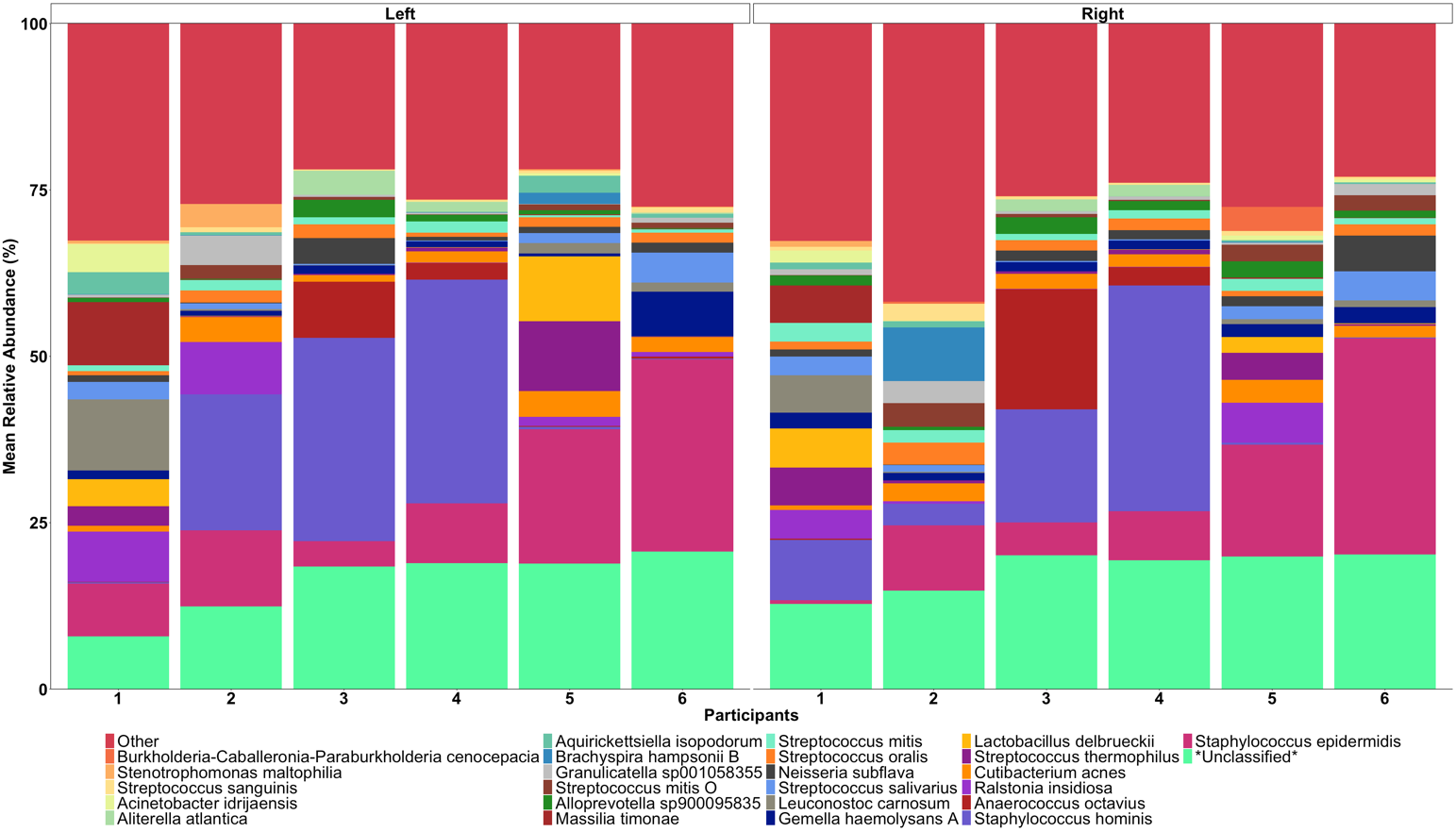
**

**Supplementary Figure 2:** Mean relative abundance bar plot of top species for left and right side of the body of each participant.
